# New Applications of Lipid and Polymer-Based Nanoparticles for Nucleic Acids Delivery

**DOI:** 10.3390/pharmaceutics13122053

**Published:** 2021-12-01

**Authors:** Adelina-Gabriela Niculescu, Alexandra Cătălina Bîrcă, Alexandru Mihai Grumezescu

**Affiliations:** 1 Department of Science and Engineering of Oxide Materials and Nanomaterials, University Politehnica of Bucharest, 011061 Bucharest, Romania; adelina.niculescu@upb.ro (A.-G.N.); alexandra.birca@upb.ro (A.C.B.); 2 Research Institute of the University of Bucharest—ICUB, University of Bucharest, 050657 Bucharest, Romania; 3 Academy of Romanian Scientists, Ilfov No. 3, 50044 Bucharest, Romania

**Keywords:** therapeutic nucleic acids, TNA delivery, lipid-based delivery systems, polymer-based delivery systems, lipid-polymer hybrid-based delivery systems, targeted delivery

## Abstract

Nucleic acids represent a promising lead for engineering the immune system. However, naked DNA, mRNA, siRNA, and other nucleic acids are prone to enzymatic degradation and face challenges crossing the cell membrane. Therefore, increasing research has been recently focused on developing novel delivery systems that are able to overcome these drawbacks. Particular attention has been drawn to designing lipid and polymer-based nanoparticles that protect nucleic acids and ensure their targeted delivery, controlled release, and enhanced cellular uptake. In this respect, this review aims to present the recent advances in the field, highlighting the possibility of using these nanosystems for therapeutic and prophylactic purposes towards combatting a broad range of infectious, chronic, and genetic disorders.

## 1. Introduction

The recent investigation of a multitude of therapeutic nucleic acids (TNAs) has paved the way for developing new treatment strategies for various wounds, infectious diseases, and chronic conditions. TNAs, such as plasmid DNA (pDNA), small interfering (or silencing) RNA (siRNA), self-amplifying RNA (saRNA), microRNA mimics, anti-microRNA oligonucleotides, messenger RNA (mRNA), and antisense oligonucleotides (ASOs), can be employed for creating therapeutics and vaccines that provide a cell-mediated immune response [1,2,3,4,5,6,7].

TNAs have gained increasing scientific interest, especially due to the long-lasting effects they offer in contrast to conventional treatments. Specifically, traditional medication induces temporary effects as it targets proteins instead of the underlying causes. In comparison, TNAs have the potential to produce long-term and even curative effects by gene inhibition, addition, replacement, or editing [2].

However, the direct delivery of nucleic acids has several drawbacks as naked nucleic acids are prone to enzymatic degradation, renal clearance, and poor cellular uptake as they face difficulties in crossing the cell membrane [4,8,9,10]. These challenges are mainly due to TNAs’ large molecular weight, negatively charged backbone, and more fragile and immunogenic potential than their oligonucleotide counterparts [6,11]. Thus, the clinical translation of such treatments is highly dependent on the delivery technologies that must enhance TNAs’ stability, protect against extracellular degradation, facilitate cell internalization, and improve target affinity [2,3,8].

In this respect, a broad range of delivery systems has been studied. Delivery vehicles such as lipid nanoparticles, polymeric micelles, dendrimers, polymer-based nanoparticles, hydrogels, polyplexes, proteins, and inorganic nanomaterials are under investigation [1,12]. Out of this plethora of possibilities, lipid nanoparticles are the most clinically advanced [11,13,14]; however, polymer and polymer–lipid hybrid particles have also recently become important categories of carrier platforms [15,16,17,18]. Moreover, such delivery systems have become highly attractive due to their targeting potential as they can be surface functionalized to allow accumulation and payload release in specific tissues [11,19,20].

In this context, this paper aims to provide an overview of the newly developed lipid and polymer-based nanosystems for nucleic acid delivery, emphasizing their importance in combatting various infectious, chronic, and genetic disorders.

## 2. Lipid-Based Delivery Systems

**Figure 1:**
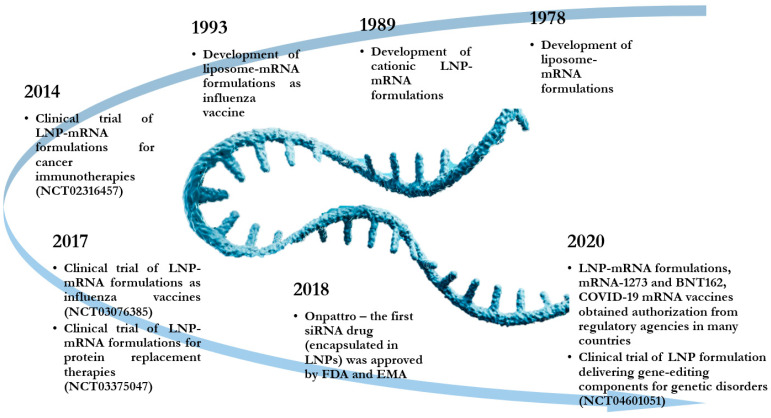
Most important benchmarks in the development of lipid-based delivery systems for RNA. Adapted from [2,11,16,24], published by Nature Publishing Group, 2021; Elsevier, 2020; Nature Publishing Group, 2021; https://www.sigmaaldrich.com/deepweb/assets/sigmaaldrich/product/documents/376/366/safc-lipids-rna-wp7004en-ms.pdf, accessed on 10 October 2021.

Lipid nanoparticles (LNPs) have attracted considerable research interest for encapsulating TNAs, especially due to their ionizable lipid presence, which is cationic at a low pH, thus, allowing complexation with negatively charged RNA or DNA [21,22]. Various generations of lipid nanocarriers have been tackled for TNAs delivery, including liposomes, solid lipid nanoparticles, nanostructured lipid carriers, and cationic lipid–nucleic acid complexes [23]. A brief history of how lipid-based RNA delivery systems evolved until the date is represented in Figure 1.

**Figure 2:**
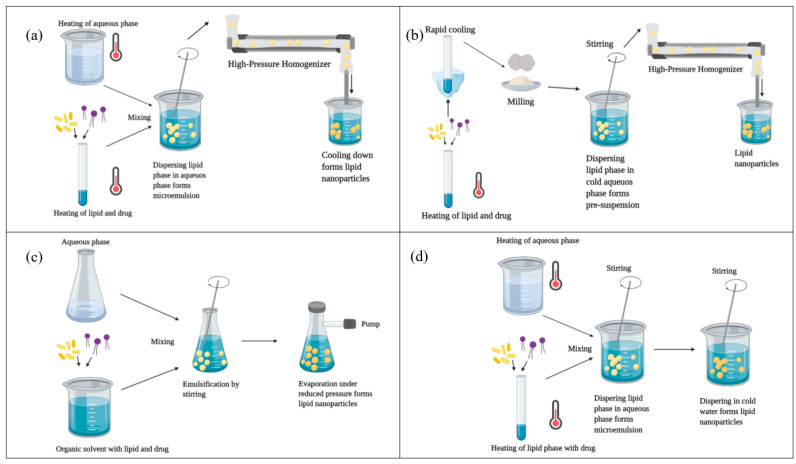
Visual representation of several conventional synthesis methods for LNP-based delivery systems. (**a**) hot high-pressure homogenization method; (**b**) cold high-pressure homogenization method; (**c**) solvent evaporation method; (**d**) microemulsion method. Adapted from [31], published by Uppsala University, 2020.

LNPs have also been extensively studied due to the relatively easy and scalable manufacturing processes they can be obtained through [3]. Specialized manufacturing and control techniques ensure the complex morphology and tailored lipid components and proportions required to create functional and efficient LNP-based delivery systems [11]. Examples of conventional synthesis methods include high-pressure homogenization [25,26], solvent emulsification–evaporation [27], ethanol injection nanoprecipitation [28], freeze-drying [29], and preformed vesicle method [30] (Figure 2).

For instance, Gomez-Aguado et al. [27] used the solvent emulsification-evaporation method to develop different solid lipid nanoparticles (SLNs) that combine cationic and ionizable lipids for the delivery of mRNA and pDNA. Their comparative study showed a higher percentage of transfected cells in mRNA formulation than for particles containing pDNA, especially in human retinal pigment epithelial cells (ARPE-19). Nonetheless, pDNA delivery resulted in greater protein production per cell in the mentioned cell line. Among the tested lipid formulations, SLNs containing only 1,2-dioleoyl-3-trimethylammonium-propane are considered the most promising for TNAs delivery. 

Other synthesis methods were used by Wang et al. [29], who have created aerosolizable dry powder of lipid nanoparticles by thin-film freeze-drying (TFFD), spray drying, and conventional shelf freeze-drying. The researchers comparatively evaluated the obtained powders and tested the feasibility of engineering SLNs using the TFFD method, concluding that this synthesis strategy leads to better aerosol performance properties than the powders obtained otherwise. 

However, some of the newest approaches for synthesizing LNPs are based on the direct mix of the organic phase (containing the lipids) with the aqueous phase (containing the TNA) using a microfluidic device [32]. Microfluidics represents a robust, scalable, and reproducible synthesis method [33]. This strategy has several advantages compared to conventional techniques as it allows the strict and easy control of flow rates, rapid mixing of the phases, and decreased synthesis time, resulting in nanoparticles with a small size, high monodispersity, and high encapsulation efficiency [28,34].

**Figure 3:**
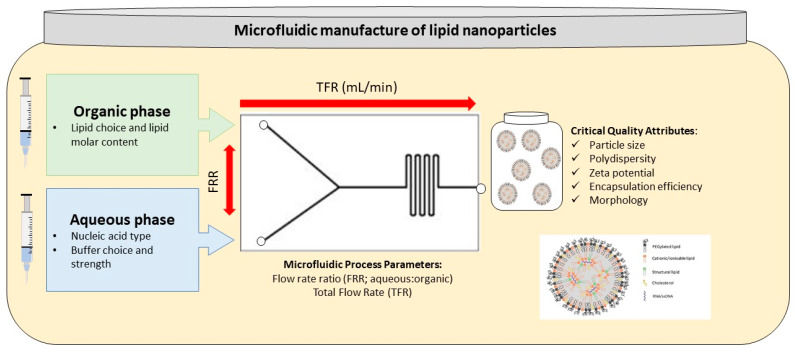
Schematic representation of LNP-based delivery systems manufacturing through microfluidic methods. Adapted from [33], published by MDPI, 2020.

For instance, Bailet-Hytholt et al. [21] have synthesized LNPs encapsulated with mRNA or pDNA by means of a precise microfluidic mixing platform. Their experimental setup allowed the self-assembly of nanoparticles under laminar flow, benefiting from advantages such as reproducibility, speed, and low volume screening. Microfluidic methods were also employed by Roces et al. [33], who have prepared PolyA, ssDNA, and mRNA encapsulated LNPs in a Y-shape staggered herringbone micromixer (Figure 3). In this manner, the researchers obtained a consistent range of particle sizes, with dimensions below 100 nm, narrow size distribution, spherical shape, good stability, and high encapsulation efficiencies.

In what concerns their applicability, numerous lipid-based TNA delivery platforms have been recently constructed as potent novel platforms for cancer immunotherapies [35]. In particular, the use of mRNA formulations for different types of solid tumors and hematological malignancies is currently examined by various studies and even clinical trials [28]. Targeted LNPs can deliver RNA-based therapeutics to leukocytes to allow the precise and modular manipulation of gene expression, thus being a highly promising approach for many immune-related disorders, such as cancer, autoimmunity, and susceptibility to infectious diseases [36].

TNAs can also be employed in formulations for the treatment and prevention of cardiovascular diseases [37,38]. Clinical trials have investigated ASOs for lowering low-density lipoprotein cholesterol (LDL-C) [39], lowering transthyretin (TTR) blood levels [40,41,42], reducing atrial fibrillation burden [43], reducing serum lipoprotein (a) levels [44], and preventing neovascular glaucoma [45]. mRNA has also been evaluated for cardiovascular conditions, with several LNP-based formulations being tested for enhancing cardiac function and regeneration [46], reducing LDL-C levels [47], and treating endotheliopathy associated with vascular senescence [48,49]. Similarly, siRNA lipid-based formulations have been clinically tested for TTR-mediated amyloidosis [50] and hypercholesterolemia [51].

**Figure 4:**
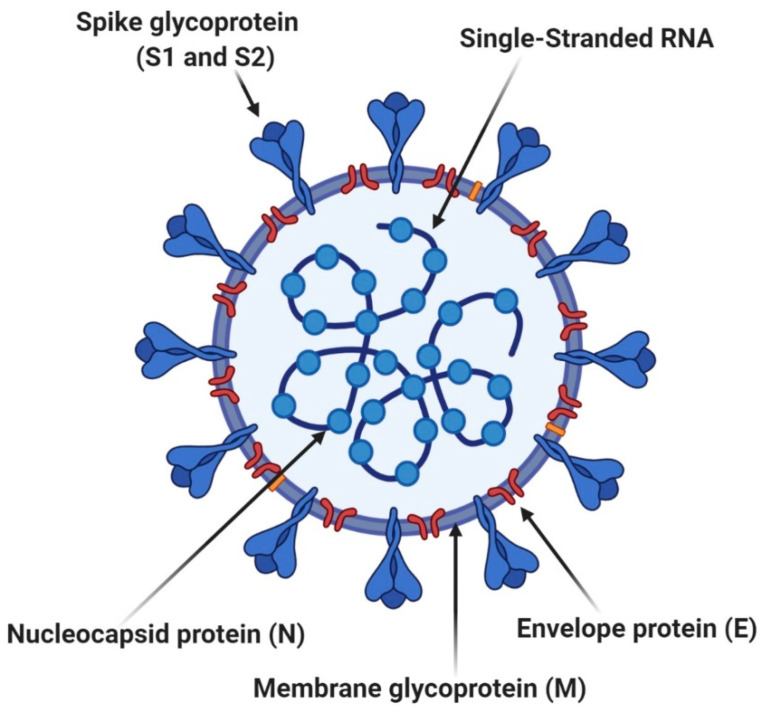
Cross-sectional model of SARS-CoV-2. Adapted from [60], published by AMER PUBLIC HEALTH ASSOC INC, 2021.

Another highly researched application of LNPs is for the development of COVID-19 vaccines, out of which several formulations have already entered clinical use for mRNA delivery [16,52,53,54,55]. Generally, these vaccines work on the principle of encoding the spike glycoprotein of SARS-CoV-2 (Figure 4) and directing the immune system against these antigens [56,57]. Clinical trials demonstrated the efficacy of LNP-mRNA formulations and an acceptable safety profile, leading to their approval for mass immunization [58,59].

COVID-19 vaccines are the most known current examples that make use of LNPs encapsulated with TNA, but this approach can be used for other infectious diseases as well [37,61,62,63,64,65,66,67]. TNA-LNP formulations have been evaluated against Zika virus [68], influenza [69,70], cytomegalovirus [71,72], chikungunya virus [73], hepatitis B [74,75,76], hepatitis C [77,78,79], human immunodeficiency virus (HIV) [80,81], and genital herpes [82]. 

The development of nonviral platforms for prenatal TNA delivery has recently emerged among the scientific research hot topics as a strategy to treat diseases before the onset of irreversible pathology and a non-invasive alternative to difficult-to-perform surgeries. What is more, the small size of a fetus compared to a postnatal recipient is a key factor for maximizing the delivery vector titer per weight of the recipient, thus, facilitating gene transduction [37,83]. In this regard, Riley et al. [84] have recently developed a library of ionizable LNPs for in utero mRNA delivery to mouse fetuses. Their LNPs successfully accumulated within fetal livers, lungs, and intestines, with higher therapeutic efficacy and safety than reference delivery systems (i.e., DLin-MC3-DMA and jetPEI), being promising candidates for protein replacement and gene editing platforms. 

A series of examples of potential applications of lipid-based TNA delivery systems have been synthesized in Table 1 to summarize the above discussion and emphasize the versatility of such formulations.

## 3. Polymer-Based Delivery Systems

Cationic polymers have attracted interest for the development of innovative TNA carriers. The main reasoning is that they form electrostatic nanocomplexes with nucleic acids, which are highly negative, to facilitate their uptake by targeted cells. Alternatively, other hydrophobic polymers can physically entrap TNAs within nanoparticles [4]. 

The first polymer tested as a vehicle for in vitro-transcribed mRNA was diethylaminoethyl (DEAE) dextran. However, after it was proven that lipid-mediated mRNA transfection is 100 to 1000 times more efficient than DEAE-dextran, the evolution of polymeric carriers has been stalled, the attention shifting towards creating perpetually improved lipid-based nanosystems [111]. 

Nonetheless, after some time, research started being oriented towards developing natural and synthetic polymer-based nanosystems for TNAs delivery as an alternative for cancer treatments and non-viral vaccine formulations. Polymers such as hyaluronic acid [94,112,113,114], chitosan [94,113], DEAE dextran [111], poly-L-lysine (PLL) [94], poly-beta-amino-esters (PBAE) [111], polyethylenimine (PEI) [94,111], dimethylaminoethyl methacrylate (DEAMA) [111], poly(ethylene glycol) methacrylate (PEGMA) [111], and DEAEMA-co-n-butyl methacrylate [111] represent promising candidates for various TNAs delivery platforms. 

Another interesting possibility is to combine two or more polymers in the same delivery system. For instance, Lü et al. [115] have developed antibody (Ab)-conjugated lactic-co-glycolic-PEI nanoparticles (LGA-PEI NPs) for the active-targeting delivery of TNAs. The research group obtained promising results for pancreatic cancer cells delivery both in vitro and in vivo, concluding that the synthesized carriers can be employed for other types of cancers as well if other specific biomarkers are targeted. 

Polymers have also been tested for microneedle-based TNA delivery [111]. Koh et al. [116] have fabricated an mRNA-loaded dissolving microneedle patch from polyvinylpyrrolidone (PVP) for cutaneous TNA administration. This RNA-patch can mediate in vivo transgene expression for up to 72 h, the transfection efficiency and kinetics being comparable to subcutaneous injection. Moreover, this innovative delivery system induced higher cellular and humoral immune responses than subcutaneous injection. A similar administration strategy was used by Pan et al. [117], who have delivered signal transducer and activity transcription 3 (STAT3)-targeting PEI-encapsulated siRNA through dextran-hyaluronic acid dissolving microneedles. The researchers obtained promising results as the microneedles effectively penetrated and rapidly dissolved into the skin. In addition, the delivered complex successfully suppressed the development of melanoma by silencing the STAT3 gene.

To briefly summarize the current research progress, several examples of polymer-based TNA delivery systems and their applications have been gathered in Table 2.

Although various types of polymers and copolymers have been tested for TNAs delivery, the poorly understood correlation between their structure and their biological response hinders their development. Moreover, the polydispersity and difficulty in metabolizing large molecular weight polymers make them less appealing than LNPs. Their clinical application is also impeded by potential toxicity, colloidal instability, and relatively poor transfection efficiency [64,111]. Therefore, more research is required for understanding the influence of polymers’ chemical structure on their biological activity and optimally modulating their properties to overcome current challenges.

## 4. Lipid-Polymer Hybrid-Based Delivery Systems

Hybrid delivery systems have recently appeared as an alternative solution for overcoming the limitations of each individual lipid or polymer component. Specifically, the adverse effects of ionizable lipids (e.g., potential immune activation, cytotoxicity) are commonly mediated by shielding the LNPs with polyethylene glycol (PEG) [11]. 

For example, Sanghani et al. [123] have PEGylated LNPs made of pH-sensitive cationic lipid CL4H6 to safely and efficiently deliver myocardin-related transcription factor B (MRTF-B) siRNA into human conjunctival fibroblasts. A similar hybrid approach on TNA delivery was taken by Scmendel et al. [20], who have developed folate-containing lipoconjugates with PEG spacers incorporated into a liposome. These delivery systems exhibited higher transfection efficiency compared to non-targeted liposomal formulations and the commercial agent Lipofectamine 2000. Improved transfection efficiency was also obtained using the hybrid nanoparticles created by Siewert et al. [124]. The researchers have developed vehicles for mRNA delivery using different proportions of the cationic lipid DOTAP and the cationic biopolymer protamine, reporting significantly increased transfection in comparison to lipid/mRNA and polymer/mRNA particles alone. Another mRNA hybrid carrier was created by Xiong et al. [125]. This research group developed theranostic dendrimer-based LNPs containing PEGylated BODIPY dyes that combine mRNA delivery and NIR imaging, holding great promise for cancer’s simultaneous detection and treatment. 

Zhou et al. [126] have synthesized a series of linear-dendritic PEG lipids (PEG-GnCm) to investigate the effect of modulating their hydrophobic domain for siRNA delivery as the surface component of dendrimer lipid-based nanoparticles (DLNPs). The researchers used different lipid alkyl lengths (C8, C12, C16) and different dendrimer generations (G1, G2, G3), creating vehicles with different physical properties and anchoring potential. These alterations did not affect particle size, RNA encapsulation, and stability but had a huge impact on delivery efficacy. PEG-G1C8, PEG-G1C12, PEG-G1C16, and PEG-G2C8 could effectively deliver siRNA in vitro and in vivo; however, all the other tested formulations lost their delivery ability, as the escape of DLNPs from endosomes at early cell incubation times was affected. 

Furthermore, by tailoring the surface charge of LNPs, they can be endowed with targeting ability. According to the comparative study performed by Gabal et al. [127], anionic nanostructured lipid carriers exhibited 1.2 times higher targeting efficiency in the brain than their cationic counterparts. Thus, such anionic particles hold promise as carriers for brain disorders therapeutics. Nonetheless, anionic formulations have not faced the same development because of difficulties encountered in nucleic acid packaging and poor transfection efficiency. To overcome these challenges, Tagalakis et al. [128] have used cationic targeting peptides as a bridge between the PEGylated anionic liposomes and the pDNA freight. The newly developed structures demonstrated improved tissue penetration, enhanced dispersal, and more widespread cellular transfection than cationic systems. Similar anionic targeting hybrid nanocarriers have also shown promising results for siRNA delivery to neuroblastoma tumors with reduced systemic and cellular toxicity [129].

Another polymer that can be used in combination with LNPs is poly(lactic-co-glycolic acid) (PLGA). In this respect, Yang et al. [130] have developed a PLGA-LNP hybrid delivery system loaded with CRISPR/Cas9 plasmids targeting the MGMT gene and modified with the cRGD peptide. This system was constructed to open the blood–brain barrier (BBB) and ensure targeted gene delivery in vivo under focused ultrasound (FUS) irradiation. The obtained results emphasized a synergic targeting ability of the physically site-specific characteristics of FUS and the biologically active targeting ability of cRGD peptide, recommending this innovative carrier as a candidate for central nervous system TNA delivery. 

**Figure 5:**
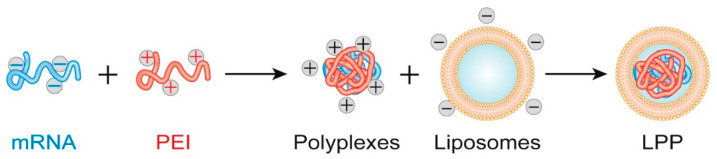
Schematic representation of lipopolyplexes formation. Adapted from [131], published by Cell Press, 2019.

A recently approached strategy for designing performant hybrid carriers is the creation of lipopolyplexes (LPPs), complexes combining nucleic acids with lipids and polymers (Figure 5). Such structures are promising delivery systems for gene therapy, especially due to their compositional, physical, and functional versatility [131,132,133]. For instance, Wang et al. [134] have synthesized a tumor-selective LPP consisting of a PEI/p21-saRNA-322 core and a hyaluronan-modulated lipid shell. The system was tested against colorectal cancer, and it was reported that it accumulated preferentially at the tumor site, leading to superior antitumor efficacy in vitro and in vivo. Alternatively, Shah et al. [135] have proposed the incorporation of ultrasound contrast agents in the liposomal cavity, followed by polyplexes addition. Thus, the scientists obtained ultrasound-activated LPPs that promote cancer cell uptake, elevate transfection efficiency, and reduce carrier cytotoxicity.

**Figure 6:**
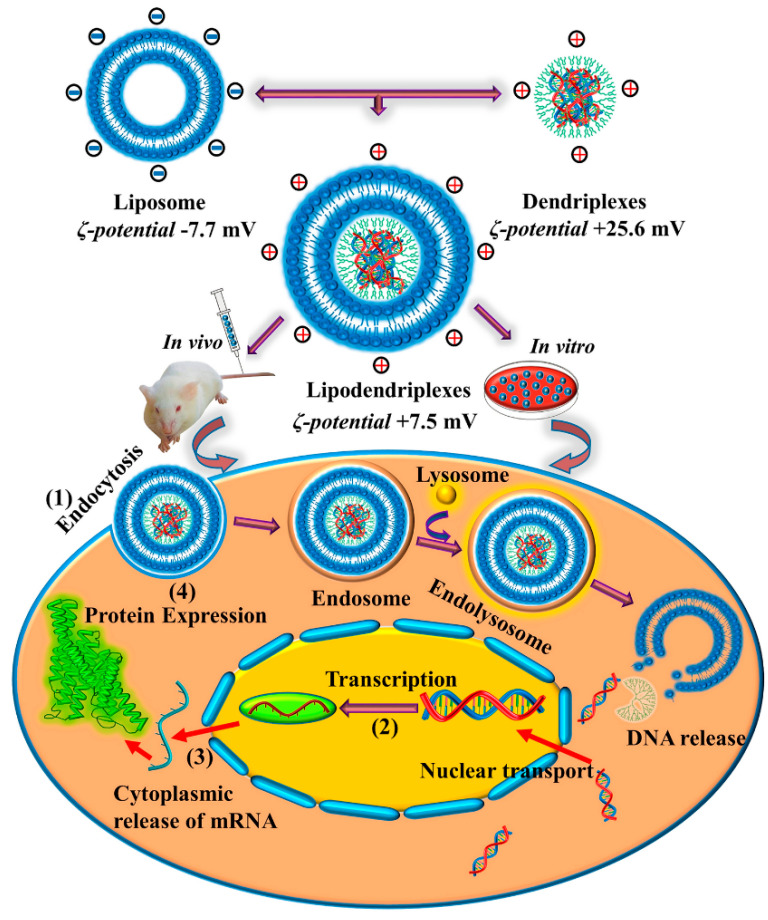
Schematic representation of lipodendriplexes formation and cellular internalization. (**1**) Endocytosis and pDNA release into the cytoplasm. (**2**) Transcription of gene-encoded DNA into mRNA. (**3**) mRNA export from the nucleus to the cytoplasm. (**4**) Protein expression. Adapted from [136], published by Nature, 2020.

One more emerging delivery possibility is TNA transport via lipodendriplexes which are complex structures formed by non-covalent hybridization of dendriplexes with the liposomal membrane. In this respect, Tariq et al. [136] have incorporated pDNA-PAMAM-based dendriplexes into an optimized liposomal formulation (Figure 6). The researchers reported significantly improved pDNA transfection, lower LDH release, lower ROS generation, higher cellular protein content, and higher cell viability compared to dendriplexes without the lipid components. Thus, these new carriers can be considered promising candidates for efficient and biocompatible gene delivery systems.

To summarize the current status of lipid polymer-hybrid-based TNA carriers, Table 3 presents several examples of such delivery systems together with their potential applications.

## 5. Challenges and Emerging Solutions to Overcome Them

LNPs are undeniably promising innovative nonviral vectors for gene delivery. Nonetheless, several challenges still exist, limiting their potential. One of the main unresolved issues is represented by the poor endosomal escape after LNP cell entry. Attempting to understand this phenomenon, Herrera et al. [140] have created a highly sensitive and robust galectin 8-GFP (Gal8-GFP) cell reporter system to visualize the endosomal escape capabilities of LNP-encapsulated mRNA. This sensor system allows the rapid and efficient distinction of endosomal membrane integrity as an indicator of cytosolic availability of mRNA. Moreover, it helped the researchers identify differences in endosomal escape capabilities elicited by the varying sterol composition of mRNA LNP-based delivery systems. 

Alternatively, Mihaila et al. [141] propose the optimization of siRNA LNP-mediated delivery by use of an ordinary differential equation (ODE)-based model. By means of mathematical modeling, the researchers designed and validated a predictive model that can compare the relative kinetics of different classes of LNPs towards choosing the best option. 

Another problem associated with LNPs is that their intravenous administration results in liver accumulation where the reticuloendothelial system takes them up. To avoid this situation, Saunders et al. [142] propose the administration of a liposome (i.e., Nanoprimer) that can temporarily occupy liver cells as a pretreatment before LNPs delivery. The researchers obtained promising results as the Nanoprimer improved the bioavailability of tested RNA-encapsulated LNPs, increased protein production, and enhanced FVII silencing.

**Figure 7:**
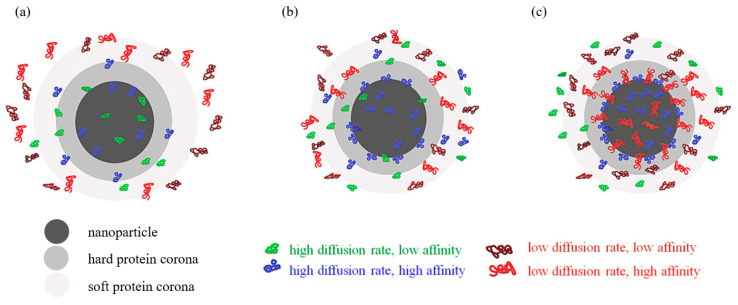
(**a**) Initiation of formation of protein corona (PC) (seconds after the NP reaches the biological fluid); (**b**) beginning of exchange from the PC of proteins with low affinity with proteins that have higher affinity (seconds to minutes); (**c**) stabilized PC, with proteins with high affinity occupying the first layer of PC (hard PC) and the majority of the second layer (soft PC) where proteins with low affinity are still present. Adapted from [146], published by MDPI, 2020.

One more aspect that must be considered regarding lipid and polymer-based TNA delivery systems is the development of a biomolecular corona around these nanoparticles (Figure 7). This “crown” is formed by electrolytes, proteins, and lipids adsorbed on nanomaterials’ surfaces when in contact with a biological environment. As the association process is almost irreversible, the biomolecular corona defines the biological identity of the nanosystem, impacting in vivo characteristics, such as circulation time, biodistribution, uptake kinetics, macrophage recognition, release profile, or targeting [143,144,145]. 

Thus, when moving from in vitro models to in vivo studies or clinical trials, it may be expected to encounter a weaker delivery efficiency and therapeutic effect of carried nucleic acids. To overcome this issue, Yang et al. [147] have proposed the design of a corona made from cyclic RGDyK peptide-modified bovine serum albumin to be used as a precoat on a redox-responsive chitosan-based nanocarrier for siRNA delivery. The synthesized corona remained steady around the delivery vehicle, improved the system’s stability, biocompatibility, and targeting ability, reduced serum proteins adsorption, increased intracellular uptake, facilitated the lysosomal escape while maintaining the redox-sensitive responsiveness of the nanocarrier.

## 6. Conclusions

To conclude, TNAs represent a promising therapeutic strategy for a broad range of diseases. Benefiting from tremendous scientific interest in the recent years, numerous LNP-based delivery systems for pDNA, mRNA, siRNA, and other nucleic acids have appeared that can help fight against various cancers, infectious diseases, cardiovascular diseases, and inherited disorders. Several such formulations have reached the clinical trials testing stage or have even been approved for use in the general population in record time, as is the case of LNP-mRNA COVID-19 vaccines. 

Other TNA delivery possibilities started to emerge as well, including polymer and lipid–polymer hybrid carriers. Nonetheless, these delivery systems are still in their infancy, most of them requiring further thorough research before moving from in vitro and in vivo tests to clinical studies. 

Overall, the evolution in developing targeted and controlled release vehicles for efficient cellular uptake faces exponentially increasing progress. Given the recent research growth in the field and the precedent it has been created with the approval of the COVID-19 vaccines, it can be expected that other TNA delivery systems will soon enter the market.

## Figures and Tables

**Table 1:** A summary of lipid-based nucleic acids delivery systems applications.

Delivery System	Physicochemical Properties	Disease/Condition	Testing Stage	Administration Route	Observations	Ref.
LNP-encapsulated C-24 alkyl phytosterols carrying mRNA	Shape: polyhedral Hydrodynamic diameter: ~100 nm Encapsulation efficiency: >90% Lipid composition: ionizable lipid (DLin-MC3-DMA, Lipid 9, or DODMA):sterol:DSPC:DMG-PEG2k at molar ratios of 50:38.5:10:1.5	Cancer	In vitro	-	High encapsulation efficiency High cellular uptake and retention Enhanced intracellular diffusivity Enhanced gene transfection	[32]
Aerosolizable siRNA-encapsulated SLNs	Size: 164.5 ± 28.3 nm Polydispersity index: 0.31 ± 0.09 Zeta potential: −29.1 ± 8.6 mV Lipid composition: lecithin, cholesterol, and lipid-PEG conjugate	Lung diseases (e.g., cystic fibrosis)	In vitro	-	SLNs diffused through the simulated mucus layer Possibility of down-regulating the expression of the gene of interest (e.g., TNF-α siRNA) by alveolar macrophages of lung epithelial cells Promising for lung delivery by inhalation	[29]
mRNA encapsulated cationic lipid-modified aminoglycosides-based LNPs	Size: 100–200 nm Surface charge: ~0 mV Encapsulation efficiency: >95% Lipid composition: GT-EP10, DOPE, cholesterol, DMG-PEG2k	Liver diseases	In vitro and In vivo (tested on mice)	Intravenous	Efficient mRNA delivery to the liver Safe delivery system (no obvious toxicity, changes in ALT or AST, or liver injury in histological sections) Potential for application in gene editing and delivery of TNA	[85]
siRNA encapsulated LNPs with different surface charges	Shape: spheres with filled cores Size: ~7- nm Zeta potential: ranged between 0 and 30 mV Encapsulation efficiency: >99% Lipid composition: lipidoid, DOTAP, DOPE, cholesterol, DSPE-PEG2k	Retinal diseases (e.g., age-related macular degeneration, glaucoma)	In vitro and In vivo (tested on mice)	Intravitreal	Successfully managed gene knockdown in mammalian cell line and primary neurons No gene silencing facilitation in the retina pigmented epithelium layer 48 h after injection, neutral and mildly positive LNPs mediated limited retinal gene suppression (<10%), whereas more positive LNPs led to approx. 25% gene suppression in the retinal ganglion cell layer	[86]
GALA-modified LNP-encapsulated pDNA	Size: 125–155 nm Zeta potential: ~15 mV (DOTMA-shell particles); ~−15 mV (YSK05-shell particles) Composition: inner coat—DOPE; STR-R8; outer coat—DOTMA/YSK05, cholesterol	Lung diseases	In vivo (tested on mice)	Intravenous	Efficient lung-selective delivery systemHigh gene expression level and significantly improved transfection activity in the lungs Improved efficiency of gene expression at the intracellular level due to the double-coating	[87]
Formulated lipidoid nanoparticles (FLNP)-encapsulated modified mRNA	Size: ~155 nm Lipid composition: epoxide-derived lipidoid complex (C14-113)	Cardiovascular disease	In vivo (tested on rats and pigs)	Intramyocardial injection	Highly efficient, rapid, and short-term mRNA expression in the heart Over 60-fold higher mRNA levels in heart tissue than for naked TNA Limited off-target biodistribution	[88]
LNP-encapsulated mRNA encoding short-lived factor VIII (FVIII) protein	Size: <100 nm RNA encapsulation: >80% Lipid composition: ionizable lipid:DSPC:cholesterol:PEG lipid, at molar ratios of 50:10:38.5:1.5	Hemophilia A	In vivo (tested on mice)	Intravenous	Safe and effective delivery platform Produced rapid and prolonged duration of FVIII expression Easy to improve FVIII function by modification of mRNA sequence encoding newly developed FVIII protein with higher activity or longer half-lifeIt could be applied to prophylactic treatment and potentially various other treatment options	[89]
Ultra-small LNPs encapsulating sorafenib and midkine-siRNA	Size: 60.47 ± 6.9 nm Zeta potential: −17.4 ± 5 mV Sorafenib encapsulation efficiency: 96.5 ± 4.8% RNA encapsulation efficiency: 94.5 ± 6.5% Lipid composition: YSK05:DOPE:cholesterol:PEG-SP94:PEG, at molar ratios of 5:2:3:1:0.3	Sorafenib-resistant hepatocellular carcinoma	In vivo (tested on mice)	Intravenous	Enhanced tumor accumulation, selectivity, and in vivo gene silencing Biosafe treatment approach Synergistic action allowed the eradication of hepatocellular carcinoma at a low drug dose (2.5 mg/kg), which is unattainable with individual monotherapy	[90]
LNP-encapsulated SARS-CoV-2 human Fc-conjugated receptor-binding domain mRNA	Size: ~100 nm Polydispersity index: <0.3 Lipid composition: ionizable lipid, DSPC, DMG-PEG, cholesterol	COVID-19	In vivo (tested on mice)	Intramuscular	Cell-free, simple, and rapid vaccine platform Produced robust humoral response Lead to a high level of neutralizing antibodies and a Th1-biased cellular response It could be used for LNP-based mRNA vaccines in general and for a COVID19 vaccine in particular	[91]
HIV-1 Env-encoded as nucleoside-modified mRNA-LNP	Size: ~80 nm Lipid composition: ionizable cationic lipid, phosphatidylcholine, cholesterol, PEG-lipid	HIV infection	In vivo (tested on rhesus macaques)	Intramuscular	Elicited durable neutralizing antibodies that were stable for at least 41 weeks Equal or better immune response when compared to adjuvanted recombinant protein vaccines	[81]
LNP-encapsulated HSV-2 nucleoside-modified mRNA	n.r.	HSV-2 infectionHSV-1 infection	In vivo (tested on mice)	Intramuscular	Potent protection against HSV-1 and HSV-2 genital infections Better at limiting virus replication in the genital tract than protein-based vaccine alternative Prevented HSV invasion of the dorsal root ganglia in 97.5% of infected mice	[82]
Atu027 (liposome-encapsulated siRNA)	Composition: positively charged AtuFect01, a neutral, fusogenic DPhyPE helperlipid and the PEGylated lipid MPEG-2000-DSPE, at molar ratios of 50:49:1	Advanced or metastatic pancreatic adenocarcinoma	Clinical trial—Phase 1/2	Intravenous	Used in combination with gentamicin-based chemotherapeutic treatment Enhanced antitumor activity Disease stabilization for 41% of patients	[92,93,94]
MTL-CEBPA (double-stranded RNA formulated into a SMARTICLES^®^ liposomal nanoparticle)	Lipid composition: liposomal formulation	Advanced hepatocellular carcinoma Cirrhosis	Clinical trial—Phase 1	Intravenous	Administered as monotherapy or in combination with sorafenib Acceptable safety profile (comparable to drugs such as sorafenib, regorafenib, and nivolumab)Antitumor activity with a mean progression-free survival of 4.6 months	[95,96]
EphA2-targeting DOPC-encapsulated siRNA	Lipid composition: DOPC	Advanced/recurrent malignant solid neoplasm	Clinical trial—Phase 1	Intravenous	Dose-escalation study Potential of slowing tumor cells growth by shutting down the causative gene	[97]
DCR-MYC (synthetic double-stranded RNA in a stable LNP suspension)	n.r.	Solid tumors Multiple myeloma Non-Hodgkins Lymphoma Pancreatic neuroendocrine tumors Primitive neuro-ectodermal tumors	Clinical trial—Phase 1	Intravenous	Dose-escalation study Antitumor potential by inhibiting MYC oncogene and thereby inhibiting cancer cell growth	[98]
mRNA-2416 (LNP-encapsulated mRNA encoding human OX40L)	n.r.	Relapsed/refractory solid tumor malignancies or lymphoma Ovarian cancer	Clinical trial—Phase 1/2	Intratumoral	Dose-escalation study Administered alone or in combination with fixed doses of durvalumab Potential immunomodulatory and antitumor activities	[99]
SGT-53 (cationic liposome encapsulating a normal human wild type p53 DNA sequence in a plasmid backbone)	Lipid composition: complex DOTAP:DOPE cationic liposome	Neoplasm Recurrent/refractory solid tumors in pediatric patients	Clinical trial—Phase 1	Intravenous	Dose-escalating study Administered alone or in combination with topotecan and cyclophosphamide Potential to significantly sensitize pediatric cancer cell lines to the killing effect of the standard chemotherapeutic agents	[100,101]
SGT-53	Lipid composition: complex DOTAP:DOPE cationic liposome	Metastatic pancreatic cancer	Clinical trial—Phase 2	Intravenous	Administered in combination with gemcitabine/nab-paclitaxel Efficient and specific delivery of p53 cDNA to tumor cells Expected to restore wtp53 function in the apoptotic pathway	[101,102]
JVRS-100 (cationic liposome-plasmid DNA complex)	Lipid composition: DOTIM/cholesterol cationic liposome	Relapsed/refractory leukemia	Clinical trial—Phase 1	Intravenous	Dose-escalation study Accelerated titration schema followed with one patient at each dose levelPotential immunostimulant activity	[103]
mRNA-1273 (LNP-encapsulated mRNA encoding for full-length perfusion stabilized spike protein of SARS-CoV-2)	Lipid composition: ionizable lipid SM-102, DSPC, cholesterol, DMG-PEG2k, at molar ratios of 50:10:38.5:1.5	COVID-19	Clinical trial—Phase 1	Intramuscular	Dose-ranging studyEvaluation of the safety and reactogenicity of a second dose vaccination schedule	[104,105]
mRNA-1273	Lipid composition: ionizable lipid SM-102, DSPC, cholesterol, DMG-PEG2k, at molar ratios of 50:10:38.5:1.5	COVID-19	Clinical trial—Phase 2	Intramuscular	Analyze the development of cellular and humoral immunity against SARS-CoV-2 after administration of the third dose of vaccine in renal or renopancreatic transplant patients who have remained seronegative after the standard two-dose regimen	[105,106]
LNP-encapsulated mRNA encoding the receptor-binding domain (RBD) of spike glycoprotein of SARS-CoV-2	Lipid composition: lipid 9001, cholesterol, DSPC, DMG-PEG2k	COVID-19	Clinical trial—Phase 3	Intramuscular	Evaluation of humoral immunity induced by the investigational vaccine and solicited adverse effects observed within 7 days post-immunization Evaluation of protective efficacy Adverse events collection over 0–28 days after each vaccination Serious adverse events collection from Dose 1 through 12 months post complete series	[107]
Comirnaty (COVID-19 mRNA-embedded in LNPs)	Lipid composition: ALC-0315, DSPC, cholesterol, ALC-0159, at molar ratios of 46.3:9.4:42.7:1.6	COVID-19	Clinical trial—Phase 4	Intramuscular	Vaccine given in two doses in patients with primary or secondary immunosuppressive disorders Results compared to a group of healthy control individuals Investigation of safety and immune responses under 6 months of time for each immunized participant	[105,108]
BNT163b2 (LNP-formulated nucleoside-modified RNA encoding for full-length perfusion stabilized spike protein of SARS-CoV-2)	Lipid composition: ALC-0315, DSPC, cholesterol, ALC-0159, at molar ratios of 46.3:9.4:42.7:1.6	COVID-19	Clinical trials—Phase 4	Intramuscular	Administration of a third vaccine dose in adults who received two doses of an inactivated COVID-19 vaccine at least 3 months prior to the study Evaluation of humoral immunogenicity, reactogenicity, and safety of a heterologous third vaccination dose	[109]
mRNA-1273.351 (LNP-encapsulated mRNA encoding for full-length perfusion stabilized spike protein of SARS-CoV-2 B.1.351 variant)	Lipid composition: ionizable lipid SM-102, DSPC, cholesterol, DMG-PEG2k, at molar ratios of 50:10:38.5:1.5	COVID-19	Clinical trial—Phase 1	Intramuscular	Evaluation of the safety, reactogenicity, and immunogenicity of the new vaccine variant Investigations on both naïve and previously vaccinated individuals	[110]

n.r.—not reported.

**Table 2:** A summary of polymer-based nucleic acids delivery systems applications.

Delivery System	Physicochemical Properties	Disease/Condition	Testing Stage	Administration Route	Observations	Ref.
Modified dendrimer nanoparticle (MDNP)-based RNA replicon	Composition: modified PAMAM dendrimer:DMPE-PEG2k:RNA at a mass ratio of 11.5:1:2.3	Zika virus infection	Ex vivo (tested on mice)	-	Tool to generate ZIKV vaccine in the absence of reference virus stocks Elicited ZIKV E protein-specific IgG responses Assessment of immune responses without the need for recombinant production of native glycoprotein	[118,119]
Ab-conjugated LGA-PEI NPs for the delivery of TNAs	Size: ~100–200 nm Composition: LGA, PEI, TNAs in various mass ratios	Pancreatic cancer	In vitro and In vivo (tested on mice)	Intravenous	Effective loading of TNAs, such as pDNA, mRNA, and miRNA, resulting in stable and functionalized nucleic acids Improved binding, internalization, and gene expression in pancreatic cancer cells with high expression of targeted cell surface markers The developed systems can be extended to other types of cancers that express specific biomarkers	[115]
STAT3-targeting PEI-encapsulated siRNA	Size: 135.1 ± 5.2 nm Zeta potential: 34.6 ± 2.2. mV N/P ratio: 10	Skin melanoma	In vitro and In vivo (tested on mice)	Intradermal	Minimally invasive administration Enhanced cellular uptake and transfection of siRNA Enhanced gene silencing efficiency Inhibited tumor cells growth in a dose-dependent manner	[117]
PEI-based nanoparticle encapsulated with IGF1 modified mRNA	N/P ratio: 6	Hypoxia Myocardial infarction	In vivo (tested on mice)	Intramyocardial injection	Potential for an extended cytoprotective effect of transient IGF1 Promoted cardiomyocyte survival and abrogated cell apoptosis under hypoxia-induced apoptosis conditions Induced downstream increases in the levels of Akt and Erk phosphorylation	[120]
CALAA-01 (cyclodextrin containing polymer encapsulating anti-R2 siRNA)	Composition: duplex of synthetic, non-chemically-modified siRNA (C05C), cyclodextrin-containing polymer (CAL101), stabilizing agent (AD-PEG), targeting agent (AD-PEG-Tf)	Relapsed or refractory cancer Solid tumors	Clical trial—Phase 1	Intravenous	siRNA-containing nanocomplexes are targeted to cells that overexpress the transferrin receptor (TfR) transferrin binds to TfRs on the cell surface, and the siRNA-containing nanocomplex enters the cell by endocytosis inside the cell, the polymer unpacks the siRNA, allowing it to function via RNA interference significant intratumoral downregulating of the target protein	[121]
siG12D-LODER	Composition: miniature biodegradable biopolymeric matrix loaded with siRNA	Pancreatic ductal adenocarcinomaPancreatic cancer	Clinical trial—Phase 2	Implantation	Highly effective and safe device implantation High safety and tolerability profiles Progression-free survival in the study population	[122]

**Table 3:** A summary of lipid-polymer hybrid-based nucleic acids delivery systems applications.

Delivery System	Physicochemical Properties	Disease/Condition	Testing Stage	Administration Route	Observations	Ref.
PEGylated CL4H6-MRTF-B siRNA-loaded LNPs	Size: ~200 nm Shape: spherical N/P ratio: 7.5 Composition: CL4HS:DOPE: PEG-DMG. At molar ratio of 50:50:1	Conjunctival fibrosis	In vitro	-	Non-toxic at a concentration of 50 nM Effectively silenced the MRTF-B gene Enhanced encapsulation efficiency Decreased fibroblast contraction after a single transfection Remarkable efficiency even after long periods of refrigeration	[123]
Ultrasound-activated LPPs	Size: ~170–250 nmComposition: polyplexes—PEI, pDNA; lipid formulation—DPPC, cholesterol, DPPG, PEG40S	Ovarian cancer	In vitro	-	Safe method for gene delivery Enhanced transfection efficiency and low cytotoxicity when exposed to low-frequency ultrasound Ultrasound exposure at a specific post-transfection time interval promotes carrier’s entry through the ECM of the cells into an intracellular environment Intracellular uptake is reported even in the presence of chlorpromazine, which seems to be the carrier’s endosomal escape	[135]
DLNPs containing PEGylated BODIPY dyes	Size: ~138 nm Zeta potential: ~−1.0 mV Composition: dendrimer-based lipid nanoparticle with BODIPY core, indole linker, and PEG-lipid of lengths between 1000 and 2000 g/mol	Cancer	In vitro and In vivo (tested on mice)	Intravenous	Particles formulated with a pH-responsive PBD-lipid produced 5—to 35-fold more functional protein than control ones formulated with traditional PEG-lipid in vitro Enhanced mRNA delivery potency in vivo Efficient functional protein expression Successfully mediated mRNA expression in tumors and simultaneously illuminated tumors via pH-responsive NIR imaging	[125]
PEGylated ionizable LNPs formulated with mRNA	Size: 60–100 nm Composition: DOPE, cholesterol, C16-PEG2000 ceramide Ionizable lipids:mRNA ratio: 10:1	Chronic liver diseases (e.g., liver fibrosis, cirrhosis)	In vitro and In vivo (tested on mice)	Intravenous	Particles obtained through microfluidic synthesis Particles were mostly distributed to the liver The highest transfection efficiency among different types of liver cells was reported for hepatocytes	[137]
siRNA-loaded LNPs conjugated with a PEG-monacyl fatty acid	Size: ~50–90 nm Zeta potential: ~ -4.3 - + 4.3 mV Composition: YSK05, DSPC, cholesterol, PEG-DMG, PEG-MO	Cancer	In vitro and In vivo (tested on mice)	Intraperitoneal injection	Significantly improved siRNA delivery efficiency as compared to originally developed LNPs (i.e., [138]) siRNA was stably retained in mouse serum, leading to a gene knockdown effect Significant gene silencing in cancer cells, ascites, and solid tumor of the mesentery	[139]
PLGA-LNPs loaded with CRISPR/Cas9 plasmids	Size: 179.6 ± 44.82 nm Zeta potential: 29.6 ± 4.33 mV Encapsulation efficiency: 76.5 ± 7.2% Composition: PLGA, lecithin, DSPE-PEG-cRGD, DSPE-PEG-biotin (lipids at a molar ratio of 7:1.5:1.5)	Glioblastoma	In vitro and In vivo (tested on mice)	Intravenous	Effective gene delivery Restored sensitivity of glioblastoma cells to temozolomide (TMZ) Highly safe and biocompatible multi-functional system Potential treatment for TMZ-resistant glioblastoma and TNA delivery to the central nervous system	[130]
Tumor-selective LPP-p21-saRNA-322	Size: 230.2 ± 10.3 nm Polydispersity index: 0.15 ± 0.02 Zeta potential: −17.3 ± 0.4 mV Composition: HA-PE tumor-selective liposomes, PEI-p21-saRNA-322 polyplexes	Colorectal cancer	In vitro and In vivo (tested on mice)	Rectal	High drug accumulation in the tumor site Efficient intracellular delivery and lysosome escapement Significant inhibition of orthotopic colorectal tumor growth Biocompatible delivery system; growth inhibition effect attributed to p21-saRNA-322 rather than the carrier	[134]
